# Combination of ^1^H NMR- and GC-MS-Based Metabonomics to Study on the Toxicity of *Coptidis Rhizome* in Rats

**DOI:** 10.1371/journal.pone.0088281

**Published:** 2014-02-05

**Authors:** Yuting Zhou, Qiongfeng Liao, Manna Lin, Xuejiao Deng, Peiting Zhang, Meicun Yao, Lei Zhang, Zhiyong Xie

**Affiliations:** 1 School of Pharmaceutical Sciences, Sun Yat-sen University, Guangzhou, People's Republic of China; 2 School of Chinese Materia Medica, Guangzhou University of Chinese Medicine, Guangzhou, People's Republic of China; Instituto de Investigación Sanitaria INCLIVA, Spain

## Abstract

**Background:**

*Coptidis Rhizome* (CR), widely applied to treat with heat and toxicity, is one of the most commonly used traditional Chinese medicine (TCM), however, an extensive dosage can induce toxicity. Diarrhea is one of the most frequent side effects of CR treatment.

**Methodology/Principal Findings:**

In this study, metabonomics was combined with the multivariate statistical analysis to discover the endogenous metabolites which related to the diarrheal induced by CR. The male Sprague–Dawley rats were dosed with 4.95 g CR/kg weight. Urine samples were collected at day −1 (before treatment), and days 14 and 21 for NMR analysis. Serum and tissues were collected at day 14 for GC-MS analysis and histopathological examination, respectively. The urine and serum metabolic profiles provided clearer distinction between CR-treated group and control group, which was confirmed by body weight change and diarrhea. Through multivariate statistical analysis, 12 marker metabolites from ^1^H NMR and 8 ones from GC-MS have been found. Among those metabolites, hippurate, acetate, alanine, glycine and glutamate are likely to break the balance of gut microbiota, whereas, lactate and 2-ketoisovalerate showed association with energy metabolism. Meanwhile, we observed that the CR-induced toxicity will recover when the treatment was stopped.

**Conclusions/significance:**

These results suggest that the main reason for the CR-associated diarrhea might be disturbance in the normal gut microbiota. This metabonomics approach may provide an effective way to study the alteration of gut microbiota, which is expected to find broader application in other drug-induced gastrointestinal reaction assessment.

## Introduction

Traditional Chinese Medicine (TCM) has gained increasing worldwide acceptance in recent years and is generally considered natural and harmless [1], [2], [3]. *Coptidis Rhizome* (CR, Chinese name is Huanglian) is a widely used Chinese herbal medicine and has been used as a heat-clearing and detoxifying agent for two thousand years. Extensive studies showed CR has many pharmacological actions with strong clinical implications, including antibacterial, anti-inflammatory, antihypertensive, antioxidative, antihyperglycemic and cholesterol-lowering effects [4], [5], [6], [7], [8], [9]. CR is relatively safe in normal dosage, however, it can also cause adverse effects. Diarrhea is one of the most frequent side effects in CR treatment, with a prevalence rate about 19% [10], while the mechanisms of CR-induced diarrhea have not been fully elucidated. In addition, CR has also been reported to cause adverse reactions such as arrhythmia and liver function injury and even lead to death in some patients, in clinic in China [11]. Therefore, it is reasonable to speculate that CR is not safe to some extent. Up to now, most studies about CR toxicity focus on the toxic components of CR and its acute toxicity. There are relatively few studies on the diarrhea associated mechanism by CR treatment.

Diarrhea usually results from increasing amounts of fluid in the intestinal lumen due to osmotically active substances (osmotic diarrhea), impairing absorption or increasing secretion of water and electrolytes (secretory diarrhea) and accelerating intestinal transit [12], [13]. Furthermore, persistent diarrhea leads to intestinal malabsorption of nutrients such as fat or bile acids and influences the composition of the microbial community. One universal activity of the intestinal microbiota is breakdown and fermentation of polysaccharides into short chain fatty acids (SCFAs) [14]. SCFAs, the major anion in stool, are synthesized from nonabsorbed carbohydrate by the colonic microbiota, interfering with the production of SCFA in the colon may result in diarrhea [15]. Alterations in the diversity of the gut microbiota are believed to underlie the development of antibiotic-associated diarrhea (AAD) [16], [17]. Therefore, we hypothesized CR-associated diarrhea may be caused by disruption of intestinal microbiota for its antibacterial activity. Hence, in this study we intend to figure out if CR induced diarrhea related to disturbance in gut microbiota and find out the biomarkers associated with gut dysbiosis to elucidate the mechanism of CR induced diarrhea.

Metabolomic investigations attempt to detect and profile changes in metabolites, which reflect changes in metabolic pathways and may provide information indicate the underlying mechanisms induced by a disease or toxic insults [18]. Nowadays, metabonomics is considered as a well-established system approach to characterize the metabolic phenotype, which results from a coordinated physiological response to various intrinsic and extrinsic parameters including environment, drugs, dietary patterns, lifestyle, genetics, and microbiome [19]. Lately, metabonomics had been successfully applied to study the mechanism of the hepatotoxicity induced by hydrazine [20] and toxicity of aconite root and its processed products [21], and to assess the associations between changes in bacterial communities' structure and dynamics of host metabolic patterns in humans [22]. In addition, as a systemic approach, metabolomics has brought enormous opportunities for improving detection of toxicity and biomarker discovery. Metabolomics reflects the function of organisms from terminal symptoms of metabolic networks and demonstrates metabolite changes of a complete system caused by interventions in a holistic context, which perfectly coincides with the holistic thinking of TCM [23], [24]. High resolution ^1^H nuclear magnetic resonance (NMR) spectrometry and gas chromatography–mass spectrometry (GC-MS)-based metabolome analysis study are well-established techniques for detection of the endogenous metabolic changes in biofluids such as serum and urine [25], [26]. Because of the fundamental differences in the nature of NMR and GC-MS measurements, the results obtained using each platform contribute supplementary understanding of the complex metabolic response of rats treated with CR [27].

The main objective of this study was to apply the histopathology examination and metabolomics to confirm whether the diarrhea induced by CR treatment results from interference in gut microbiota. ^1^H NMR and GC-MS were used to analyze the small molecular metabolites in the urine and serum samples. Multivariate data analysis, such as principal components analysis (PCA) and partial least squares-discriminant analysis (OPLS-DA) were utilized to find out related biomarkers, which could indicate the biological response to CR treatment. Our results showed that metabonomics approach may provide an effective way to study the alteration of gut microbiota.

## Materials and Methods

### Ethics statement

All animal experiments were conducted at the Sun Yat-sen University Animal Experiment Center (Guangzhou, China). The animal experiments were reviewed and approved by the Institutional Animal Care and Use Committee of Sun Yat-sen University (SYXK, Licence No. 2011-0112), and conformed to the National Institute of Health guidelines on the ethical use of animals. All surgeries were operated under sodium pentobarbital anesthesia. Because this experiment was aim to investigate the toxicity of Coptidis Rhizome in rats, we did not stop the experiment when we observed the animals were beginning to loss of body weight & diarrhea. However, considering the suffering caused by loss of body weight & diarrhea, we stopped CR treatment after 14 days of CR administration. During the 14-day administration and 7-day recovery, no animal died as a result of CR treatment and all rats were sacrificed by sodium pentobarbital anesthesia. During the period of recovery, we found that the rats began to stop diarrhea gradually after three days of recovery. Besides, the growth of rats also returned to normal level.

### Materials and chemicals

*Coptidis Rhizome* was purchased from Zhixin Medicine Health co., Ltd (Guangzhou, China), and identified by Prof. Ling Jiang (School of Pharmaceutical Sciences, Sun Yat-sen University). D_2_O (with 0.05% sodium 3-trimethylsilyl-(2, 2, 3, 3-^2^H_4_)-1-propionate, TSP), Bis (Trimethylsilyl) -Trifluo-roacetamide (BSTFA, with 1% Trimethylchlorosilane, TMCS) and heptadecanoic acid were purchased from Sigma-Aldrich. Pyridine and chloroform were GC grade from China National Pharmaceutical Group Corporation (Shanghai, China).

### Preparation of *Coptidis Rhizome* decoction

The dried roots of *Coptidis Rhizome* were ground into powder (about 30 meshes). The powder (200 g) was dipped in 2 liters of water for 1 h and then decocted to boil for 1 h. The decoction was filtered through four layers of gauze. Next, the residues were boiled once again for 1 h with 1.6 liters of water and the decoction was filtrated out with the above method. All filtrates were merged and concentrated to a volume equivalent 0.495 g *Coptidis Rhizome*/ml and stored at 4°C.

### Animal treatment and sample collection

Male Sprague-Dawley (SD) rats with specific pathogen free (SPF) (weight: 180–200 g) were purchased from the medical laboratory animal center of Guangdong province (Guangzhou, China) and allowed to acclimate to the new environment for 7 days prior to experiment in a standard experimental room (12 h light/dark cycle, 24°C and 50%–70% humidity). All animals had ad libitum access to food and water. After acclimatization for 7 days, all rats were randomly divided into two groups (control group and CR-treated group). Controls were subjected to oral gavage with distilled water, while the CR-treated group was orally administered CR water solution at a dose of 4.95 g *Coptidis Rhizome*/kg of body weight for 14 days, respectively. The dosage of 4.95 g *Coptidis Rhizome*/kg weight was determined with reference to the Chinese Pharmacopoeia (10 g per human per day) and our pre-study results.

**Figure 1:**
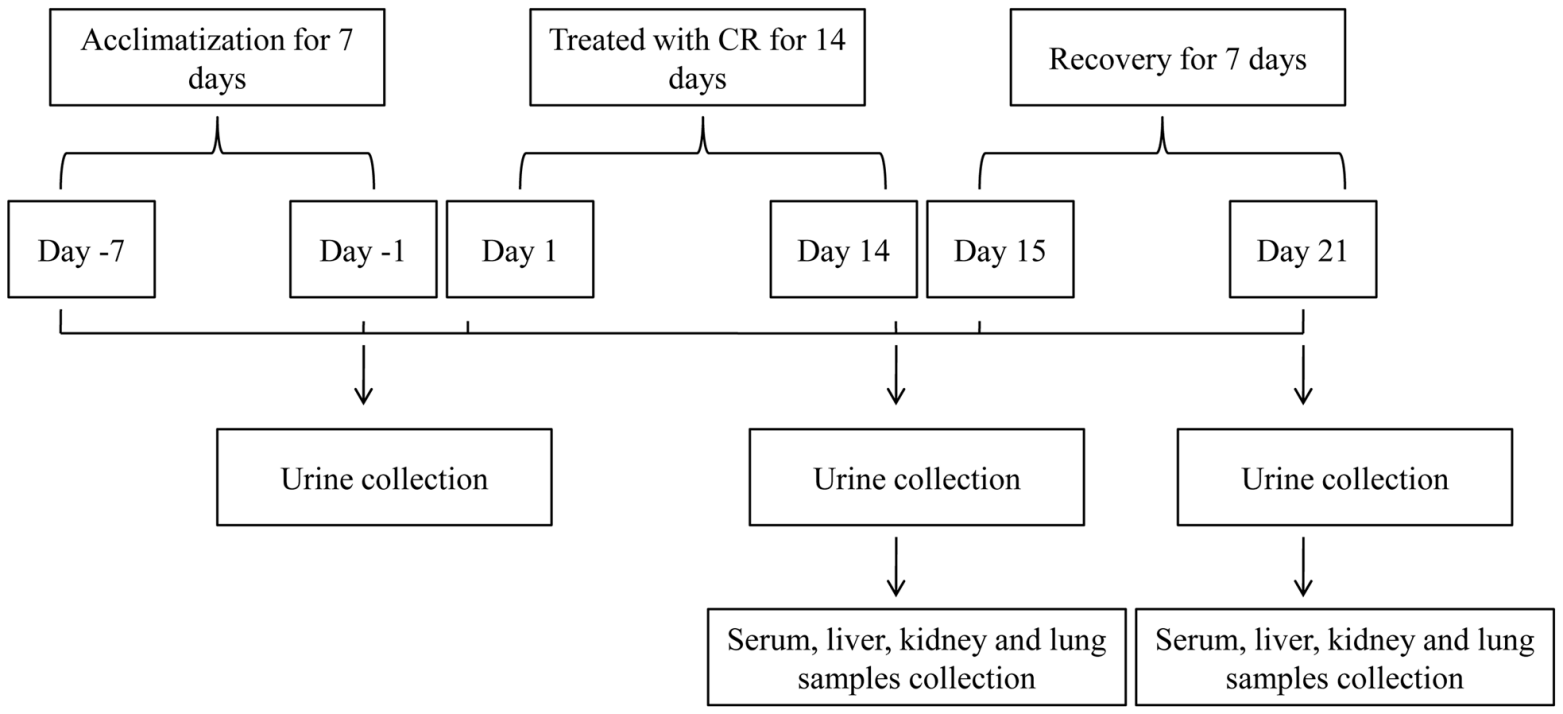
Schematic representation of animal treatment and sample collection. The experiment lasted 21 days, including 14 days of treatment with CR and 7 days of recovery, urine sample, serum sample and tissues were collected after 14 days of treatment and 7 days of recovery.

The experiment lasted 21 days, including 14 days of treatment and 7 days of recovery. Body weights were measured every day just before CR administration. The animals were observed daily for abnormalities of general appearance and behavior. Urine samples were collected from the metabolic cages with an ice packed Eppendorf tube on pre-dose day 1 and on days 14 and 21 post-dose. The fresh urine samples were immediately centrifuged at 10,000 g for 10 min, 4°C and the supernatants were collected and stored at −80°C for subsequent analysis. At the end of 14^th^ day, five rats of each group were sacrificed by sodium pentobarbital anesthesia, intraperitoneal injection. Blood was obtained from the abdominal aorta and collected in tubes without heparin in order to collect the serum. The liver, kidney, lung and heart specimens were removed and immediately fixed in 10% neutral buffered formalin for histopathological studies. The remaining rats were fed for another 7 days to recover and sacrificed at day 21 to collect serum sample and tissue specimens. The serum samples were stored at −80°C until analysis. A schematic presentation of animal treatment and sample collection was shown in Figure 1.

### Histological examination

After fixation for 48 hours, all tissues were embedded in paraffin wax according to routine procedures. Five µm thick sections were cut and stained with hematoxylin-eosin (H&E) for histopathological evaluation. Two expert testers at the department of Pathology of Sun-yat-sen University analyzed the tissue slices.

### Sample preparation and analysis

For NMR analysis, urine samples were removed from −80°C storage and thawed at room temperature, 500 µL of urine was mixed with 100 µL of phosphate buffer (0.2 M Na_2_HPO_4_/NaH_2_PO_4_, pH 7.4) to minimize chemical shift variations and then centrifuged (14,000×g, 10 min, 4°C) to remove any precipitates. The supernatant was then pipetted into 5 mm NMR tube and 80 µL of D_2_O containing 0.05% sodium 3-trimethylsilyl-(2, 2, 3, 3-^2^H_4_)-1-propionate (TSP) was added.

All ^1^H NMR spectra of urine were collected at 298 K on a Bruker Avance 500 MHz spectrometer equipped with a Bruker inverse probe. The acquisition parameters were essentially refer to those previously reported [28], [29]. The NMR spectrum was recorded using the water-presaturated standard one-dimensional Carr–Purcell–Meiboom–Gill (CPMG) pulse sequence, which can eliminate interference by macromolecules. The 90° pulse length, varying from 14.44 to 15.35 µs, was adjusted for each sample individually. 64 transients were collected into 128 k data points using a spectral width of 10 kHz with a relaxation delay of 3 s, and relaxation time (2 nτ) of 100 ms. The free induction decays (FIDs) were multiplied by an exponential function with a line-broadening factor of 0.3 Hz before Fourier transformation. The ^1^H NMR spectra were corrected for phase and baseline distortion using software MestreNova (http://www.mestrelab.com/software/).

For GC-MS analysis, Serum metabolites were subjected to trimethylsilyl derivatization and analyzed by GC-MS according to Wei Jia [30]. Briefly, internal standard solution (10 µL of L-2-chlorophenylalanine in water, 0.3 mg/mL) was spiked into 100 µL aliquot of serum sample. A mixture of methanol/chloroform (3∶1) (300 µL) was used to extract the metabolites from the serum. After a vortexing period of 30 s and storage at −20°C for 10 min, the samples were centrifuged at 10000 g for 10 min. An aliquot of the 300 µL supernatant was vacuum-dried at room temperature in a glass vial. The residue was derivatized with 80 µL of methoxyamine (15 mg/mL in pyridine) at 30°C for 90 min, and followed by 80 µL of BSTFA (1%TMCS) at 70°C for 60 min, then centrifuged at 12 000 g for 10 min.

Each 1-µL of derivatized solution was injected into a DB-5MS capillary column (30 m×250 µm i.d., 0.25 µm film thickness; Agilent J&W Scientific, Folsom, CA) and conducted on a Finnigan gas chromatograph (Thermo Fisher Scientific Inc., U.S.A.) coupled with a mass spectrometer (TRACEDSQ). Helium was used as the carrier gas at a constant flow rate of 1.0 mL/min. The injection and transfer interface temperatures were both set to 280°C. Electron impact ionization at full scan mode (m/z 50–500) was used. Source temperature, electron energy, and solvent delay were set at 230°C, 70 eV, and 5 min, respectively. The GC oven temperature programming was started at 100°C and maintained for 5 min, followed by 20°C/min ramps to 170°C and maintained at 170°C for 1 min, 10°C/min to 270°C and maintained at 270°C for 1 min, and 2°C/min to 280°C, and a final 6 min maintenance at 280°C.

### Data Processing

^1^H NMR spectra data was analyzed using the established method of spectral binning and the new technique of targeted profiling [31]. Spectral binning was performed using the software MestreNova (http://www.mestrelab.com/software/). The chemical shifts of spectra were referenced to the TSP at δ 0.00. Each NMR spectrum was reduced to 0.04 ppm-wide segments between δ 0.5 and δ 9.5 ppm. The spectral regions of water (δ 4.60–5.20 ppm) and urea (δ 5.52–6.00 ppm) were removed from the analysis for all groups in order to prevent variation in each sample. The resonance from the NMR internal standard and reference (TSP) was also excluded from the dataset prior to analysis. Each NMR variable was normalized to the total area in order to allow a spectrum-to-spectrum comparison. Bins that showed significant differences between CR-treated group and control group were then assigned to the corresponding metabolites by comparing chemical shifts and multiplicities of peaks to the literature or online databases.

Raw GC-MS files were exported into the platform-independent net CDF (*.cdf) and loaded into XCMS software (version 1.6.1) based on R-program version 2.4.0 (R-Foundation for statistical computing, www.Rproject.org), where peak peaking, integration and alignment in the time domain were performed. Integrated intensities of each m/z-retention time pair (MZRT) were obtained for each one of the samples used in the study. NIST mass spectral databases were used for metabolite identification.

### Statistical analysis

Both univariate and multivariate statistical analyses were applied using SPSS 17.0 software (SPSS, Chicago, USA) and SIMCA-P+ 12.0 (Umetrics, AB, Umeå, Sweden), respectively. Data of body weight gain and the ratio of body weight (weight gain to weight at week 0) were expressed as the mean ± standard deviation. Student's t-test was used for comparison of gain of body weight and the ratio of body weight. Significant difference was defined at p<0.05. In the multivariate statistical analyses, all normalized ^1^H NMR and GC-MS spectral data were imported to SIMCA-P+ 12.0 for principal components analysis (PCA), and orthogonal partial least-squares discriminant analysis (OPLS-DA). PCA, an unsupervised multivariate statistical approach, was performed to achieve the natural inter-relationship (grouping, clustering, and outlier detection) among samples. To maximize class discrimination, the OPLS-DA results were further analyzed. Criteria of quality of modeling were R^2^X, the cumulative modeled variation in metabolite data, R^2^Y, the cumulative modeled variation in class values, and Q^2^, the cumulative predicted variation in metabolite data according to cross-validation (7-fold cross validation). These parameters range between 0 and 1, where 1 indicates a perfect fit. Metabolites responsible for the separation between classes in supervised models were identified based on the Variable Influence on Projection (VIP) values, which correspond to the importance of the variables for the model. The variables with a VIP value larger than 1.0 were considered significant and used for further analysis and identification of the responsible peak(s) within the spectrum.

## Results

### Body weight

**Figure 2:**
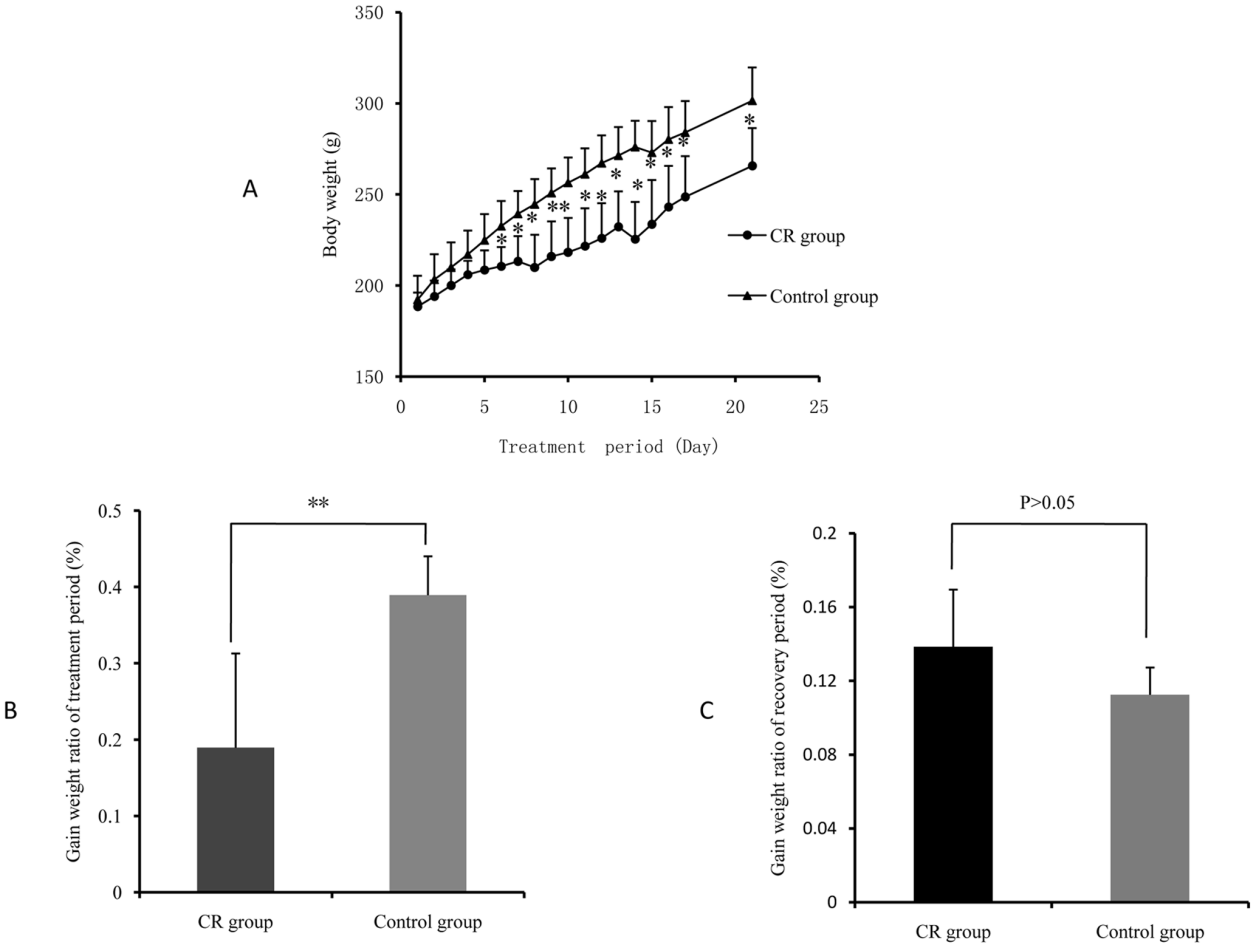
Changes of Body weight and increased body weight ratio of rats in CR-treated group and control group. A, body weight of rats in the whole experimental period; B, increased body weight ratio of treatment period; C, increased body weight ratio of recovery period. The data are expressed as means ± SD (*P<0.05; **P<0.01).

During the 14-day administration and 7-day recovery, no animal dead, but rats in the CR-treated group began diarrhea after 5 days of treatment. To investigate the toxic effects of CR, growth was firstly examined. The representative change tendency of body weight during the experiment period is showed in Figure 2A. The body weights of rats in CR-treated group were significantly lower than those in control group after 14 days of CR treatment. Furthermore, the ratio of body weight (weight gain to weight at week 0) of CR-treated group is approximately 20% lower than control group (Figure 2A and 2B). However, the ratio of body weight was no significant difference during recovery period (Figure 2C).

### Histopathology

**Figure 3:**
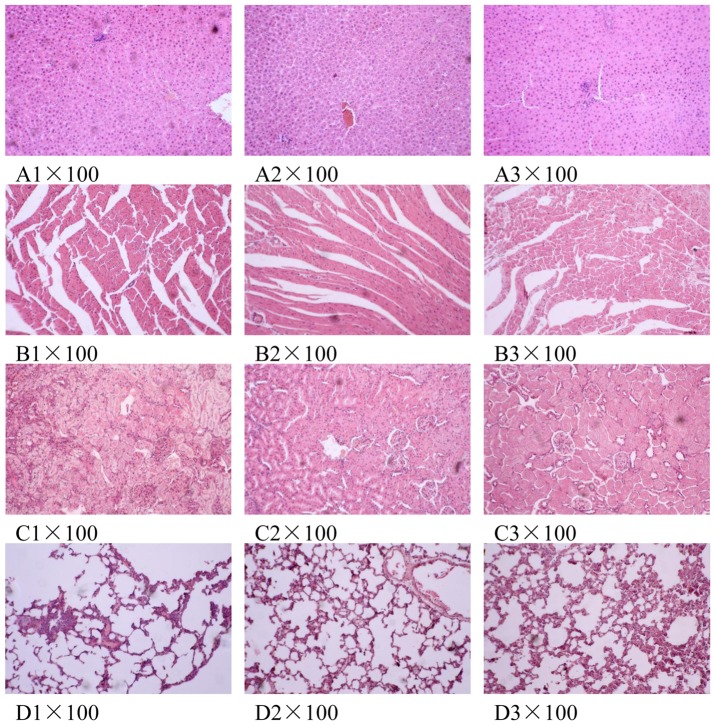
Histopathology of liver (A), heart (B), kidney (C) and lung (D) tissues. The tissues were collected after 14 days of CR treatment and 7 days of recovery. The tissues were fixed in 10% formaldehyde, and then stained using Hematoxylin and Eosin (H&E). Original magnification×100. A1, B1, C1 and D1, controls group of day 14; A2, B2, C2 and D2, CR-treated group of day 14; A3, B3, C3 and D3, CR-treated group after 7 days of recovery.

To examine the toxicity at the tissue, we analysed the histopathological data on liver, kidney, lung and heart tissues. H&E staining of liver, kidney, lung and heart tissues revealed that there was no apparent toxicity at these tissues by CR treatment. After CR administration of 14 days, three rats in CR-treated group and one in control group had only a slight morphological variation in liver (Figure 3B), in which less than 10% of cells showed infiltration or edema. Therefore, we considered this as the normal organic change in liver. The hepatic lobules in other rats had normal cell shapes. In addition, the hepatic lobular structure and portal tract were not inflammatory or hydropic (Figure 3A). There was no obvious variation in liver tissue after recovery of 7 days compared with controls (Figure 3C).

### NMR Spectroscopy of urine samples

**Figure 4:**
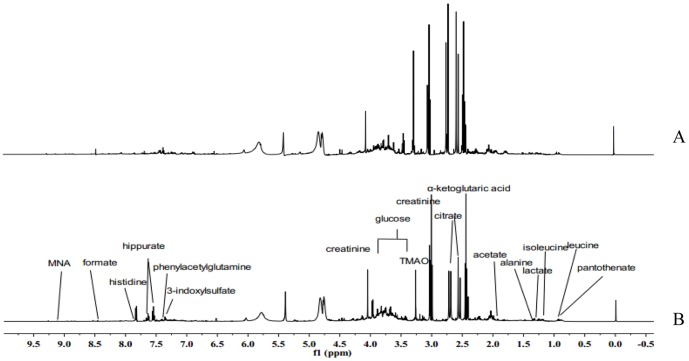
Representative urine ^1^H NMR spectra from control and CR treated rats after 14 days of treatment: A, CR-treated group; B, control group. Assignments of endogenous metabolites were based on comparing chemical shifts and multiplicities of peaks to the literature as well as the Metabonomics Toolbox (http://www.hmdb.ca). The spectra were taken for urine samples containing 0.2 M Na_2_HPO_4_/NaH_2_PO_4_, pH 7.4 and 0.05% TSP as a chemical shift reference.

**Figure 5:**
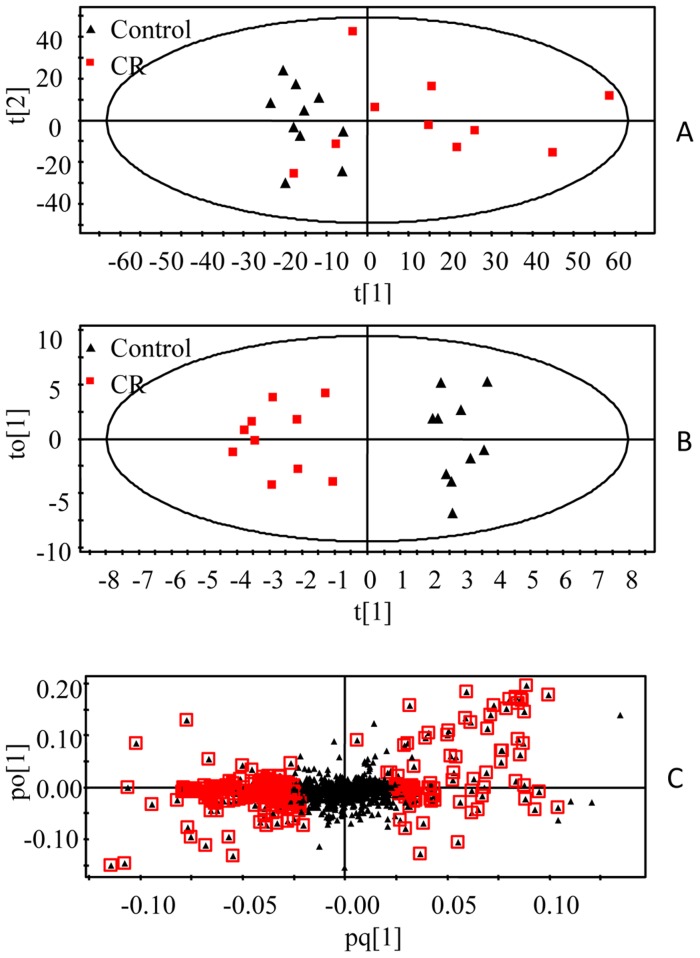
Multivariate analysis of urine samples from CR and control groups. A, PCA score plot, B, OPLS-DA score plot, C, OPLS-DA loadings plot. The urine samples were collected after 14 days of treatment. The model was established using one predictive and one orthogonal component.

Representative ^1^H NMR spectra of urine samples collected on the 14^th^ day from different group are shown in Figure 4. Assignments of endogenous metabolites involved in ^1^H NMR spectra were based on comparing chemical shifts and multiplicities of peaks to the literatures [32], [33], [34], [35] as well as the Metabonomics Toolbox (http://www.hmdb.ca). Due to the high information content and complexity of the spectra, multivariate statistical analyses were applied to reveal the metabolic changes-related CR treatment. Initially, the unsupervised principal component analysis (PCA) was applied to explore correlations between control group and CR-treated group, and a tendency to separate the two classes was detected in the score plot (R^2^X = 0.721, Q^2^ = 0.526) (Figure 5A). Since we observed intra-group variation, the supervised method, OPLS-DA, was also used to isolate the variables responsible for the differences observed between the CR and control groups (R^2^X = 0.451, R^2^Y = 0.921, Q^2^ = 0.783) (Figure 5B). The model showed an overall goodness of fit. To high light metabolite differences between the control group and CR-treated group, feature selections were performed using loadings plot, followed by OPLS-DA (Figure 5C). VIP analysis showed the order of metabolites according to their critical influence on clustering. The points marked red in Figure 4C showed the metabolites which VIP value more than 1.00. Some of these segments were found to be from the same metabolite. The key metabolites, which were essential for distinguishing between the CR and control groups were selected from the results of the VIP analysis (VIP>1.00) and Student's t-test (P<0.05). After merging the variables from the identical metabolites, 12 metabolites were collected and considered as the urine potential biomarkers (Table 1).

**Table 1:** Endogenous metabolites selected as biomarkers in urine profile and their change trends.

Metabolites	Chemical shift(ppm)	CR vs. control
2-Keto-isovalerate	1.25	↑*
Lactate	1.33	↑*
Alanine	1.46	↑*
Acetate	1.91	↑**
N-acetylglycoprotein	2.03	↑*
Glutamate	2.36	↑**
Trimethylamine	2.82	↑*
Creatine	3.00	↓*
Phenylacetylglulamine	7.41	↑**
Hippurate	7.53,7.62,7.83	↓**
Trigonelline	8.07	↓*
Formate	8.46	↓**

↑, the increase in signal;↓, the decrease in signal.

*Key: p<0.05.

**Key: p<0.01.

### GC-MS spectrometry

**Figure 6:**
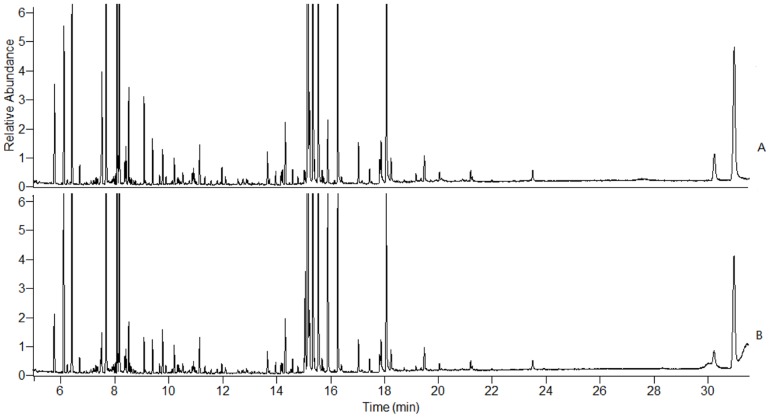
Representative GC-MS total ion chromatograms from the serum samples of control and CR treated rats after 14 days of treatment: A, control group; B, CR-treated group.

**Figure 7:**
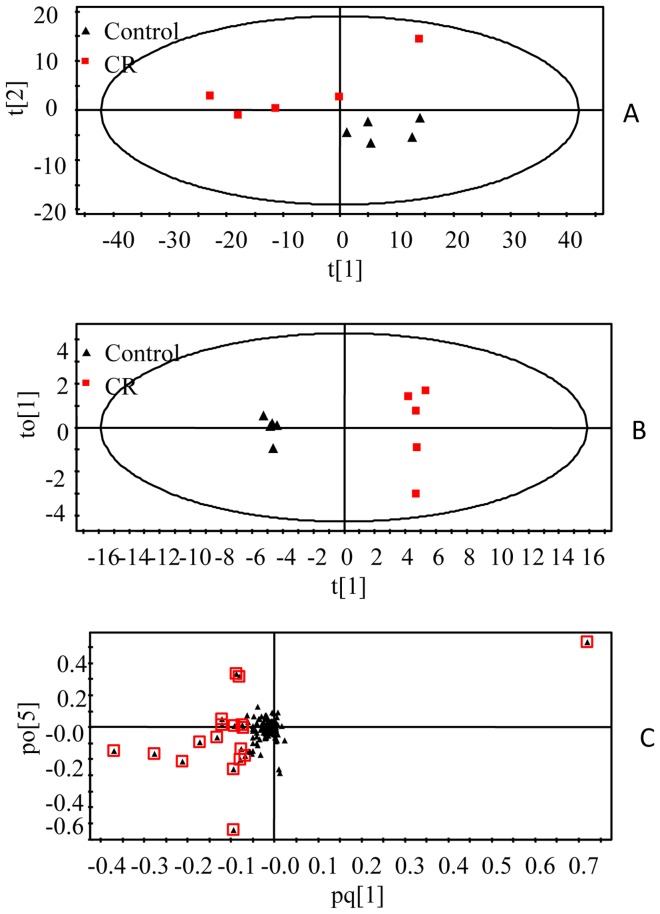
Multivariate analysis of serum samples from CR and control groups. A, PCA score plot, B, OPLS-DA score plot, C, OPLS-DA loadings plot. The urine samples were collected after 14 days of treatment. The model was established using one predictive and one orthogonal component.

Serum samples were analyzed by GC-MS. Figure 6 shows the typical total ion chromatograms (TICs) of serum samples collected on 14^th^ day. The raw GC-MS data from metabolic profiling were pretreated following the procedure in Section 2.7. The XCMS output data containing 536 ion peaks was preprocessed using the Microsoft Excels of software, where the IS (internal standard) peak, and impurity peaks from column bleeds and derivatization procedures were excluded, and the variables presenting in at least 80% of either group were extracted. Then, the remaining ion features were normalized to the internal standard. The most abundant fragment ion with the same retention time (the time bin is 0.01 min) was remained and the other ions were excluded. The processed data was further subjected to statistical analysis. In the PCA scores plot (Figure 7A), the CR-treated group and control group can be well distinguished (R^2^ = 0.864, Q^2^ = 0.687). As could be observed in the OPLS-DA score plot (Figure 7B), separation between these two groups was also clearly seen (R^2^X = 0.991, R^2^Y = 0.995, Q^2^ = 0.888), indicating that biochemical perturbation significantly happened due to CR treatment. The model showed an overall goodness of fit. According to the loadings plot (Figure 7C) of this OPLS-DA model, and using the above-stated statistically significant threshold, 8 metabolites with VIP-values greater than 1.0 and P values less than 0.05 were finally revealed to be significant in differentiating the CR treated and control groups. Alanine, L-proline, glycine, hisoindol, serine, glutamate and L-ornithine were elevated in CR-treated group, while D-glucose was reduced in this group (Table 2).

**Table 2:** Endogenous metabolites selected as biomarkers in serum profile and their change trends.

Metabolite	RT(min)	CR vs. control
Alanine	5.74	↑*
L-proline	8.4	↑*
Glycine	8.5	↑*
Hisoindol	8.55	↑*
Serine	9.08	↑*
Glutamate	11.95	↑*
L-ornithine	14.16	↑*
D-Glucose	15.14	↓*

↑, the increase in signal;↓, the decrease in signal.

*Key: p<0.05.

### Toxic effects of CR based on metabolite profiling

**Figure 8:**
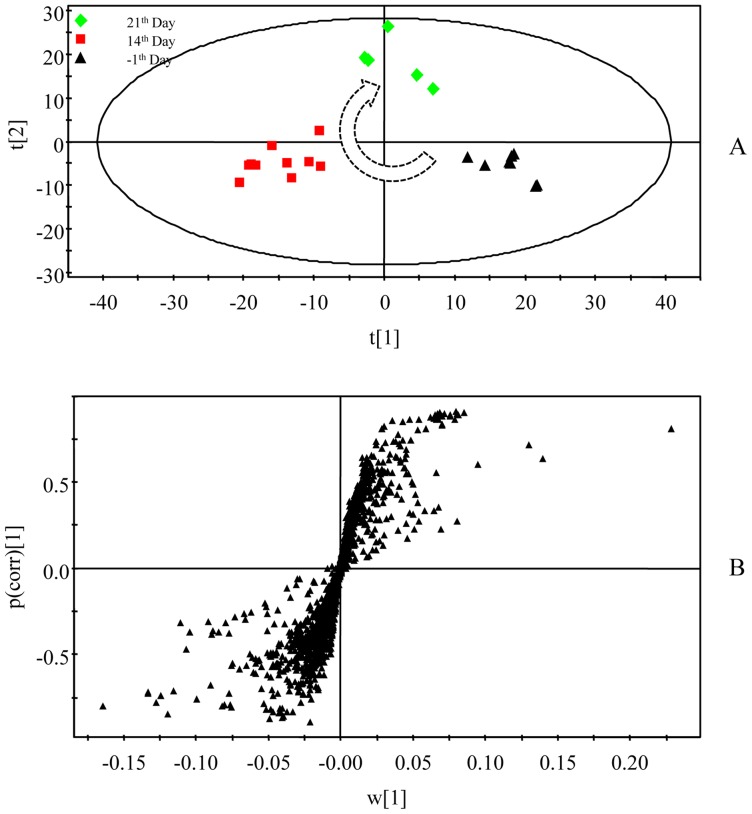
Multivariate analysis of urine samples of pre-treatment, after 14 days of treatment and after 7 days of recovery. A, OPLS-DA score plot, B, OPLS-DA loadings s-plot.

**Figure 9:**
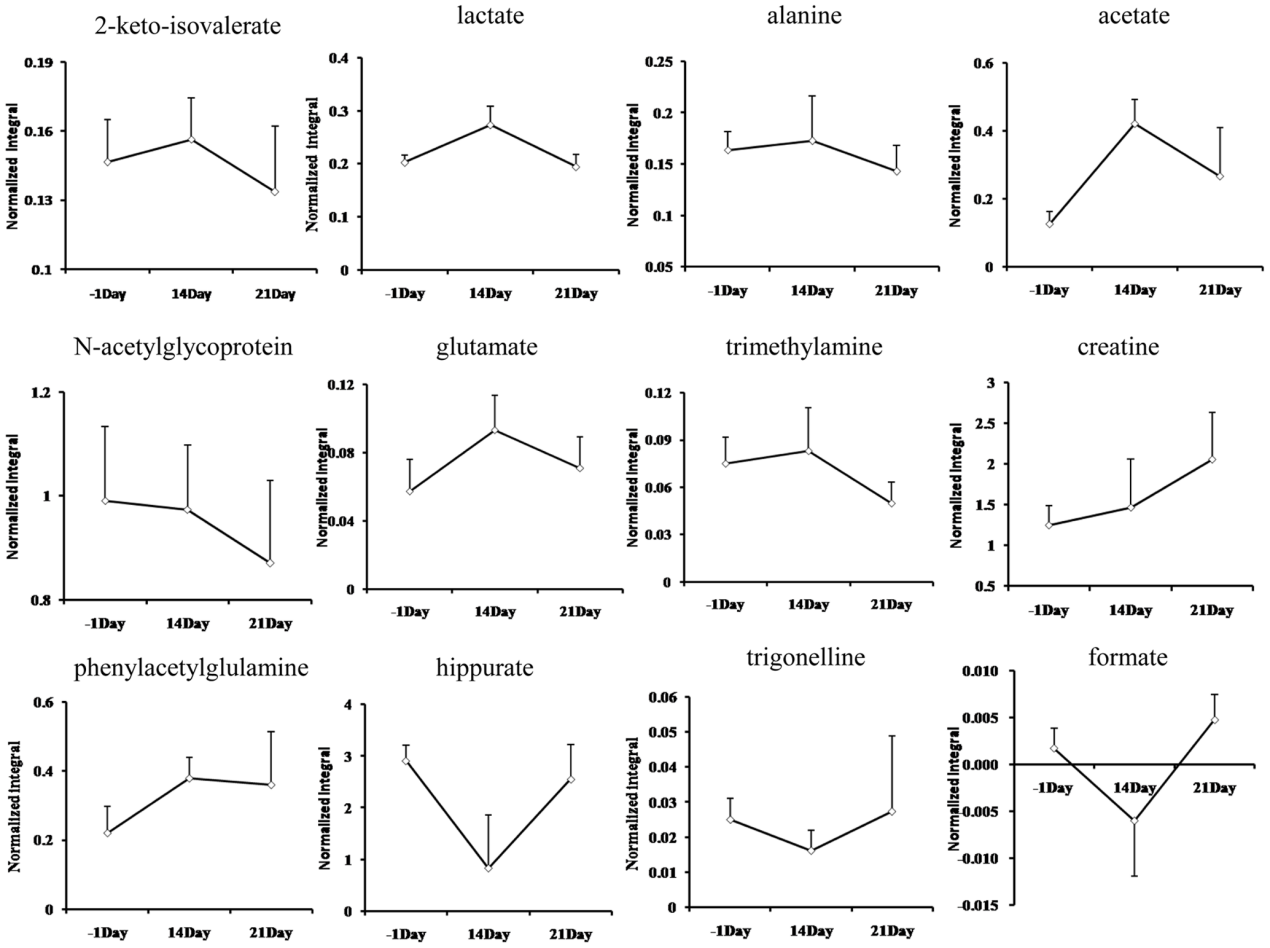
The normalized integral changes of the relevant time-related urinary marked metabolites induced by CR treatment.

Using the presented ^1^H NMR and GC-MS method, the urinary and serum metablic profiles of CR -treated group were obtained. The effects of inhibition of growth on CR-treated rats have been shown in Figure 2. However, after 7 days of recovery, the growth of rats returned to a normal rate. The diarrheal of rats also stopped after days of recovery. As the 12 potential biomarkers from ^1^H NMR and 8 ones from GC-MS have been found, it is reasonable to take them as the potential biomarker for further investigating the intervening mechanisms of CR toxicity. Therefore, the metabolic profiling of ^1^H NMR on the 21^th^ day was introduced as variables to OPLS-DA. Consequently, the ^1^H NMR data of the −1^th^, 14^th^ and 21^th^ day were analyzed using OPLS-DA. As shown in OPLS-DA scores map derived from ^1^H NMR metabolic profile (Figure 8), the 21^th^ day group is closer to the −1^th^ day group, which indicated the CR-induced toxicity tend to recover. To further evaluate the reversed condition of the potential biomarkers when CR treatment was stopped, the normalized integrals of the changes in these endogenous urinary metabolites were investigated. Figure 9 demonstrated the time-related response of these metabolites to CR treatment. Similar to the results of body weight change, the ^1^H NMR analysis presented in Figure 9 shows that 11 of the 12 detected metabolites also showed a return toward pretreatment values after 7 days of recovery.

## Discussion

CR is relatively safe in normal dosage. However, it reportedly can cause adverse effects such as sicchasia, diarrhea, respiratory failure and extrapyramidal system reactions in some patients, in clinic in China [36]. Our results were similar to those of clinical study in which people treated with CR. During the period of CR treatment, the most obvious toxicity effect of CR treatment was diarrhea. In addition, our results showed there was a significant decrease in the body weight gain of CR-treated group at during the period of CR treatment compared with the time-matched control. Further, to investigate the mechanisms underlying the toxicity of CR treatment in rats, we compared the urinary and serum metabolic profiles of rats treated with CR decoction and distilled water using ^1^H NMR and GC-MS based metabonomics.

**Figure 10:**
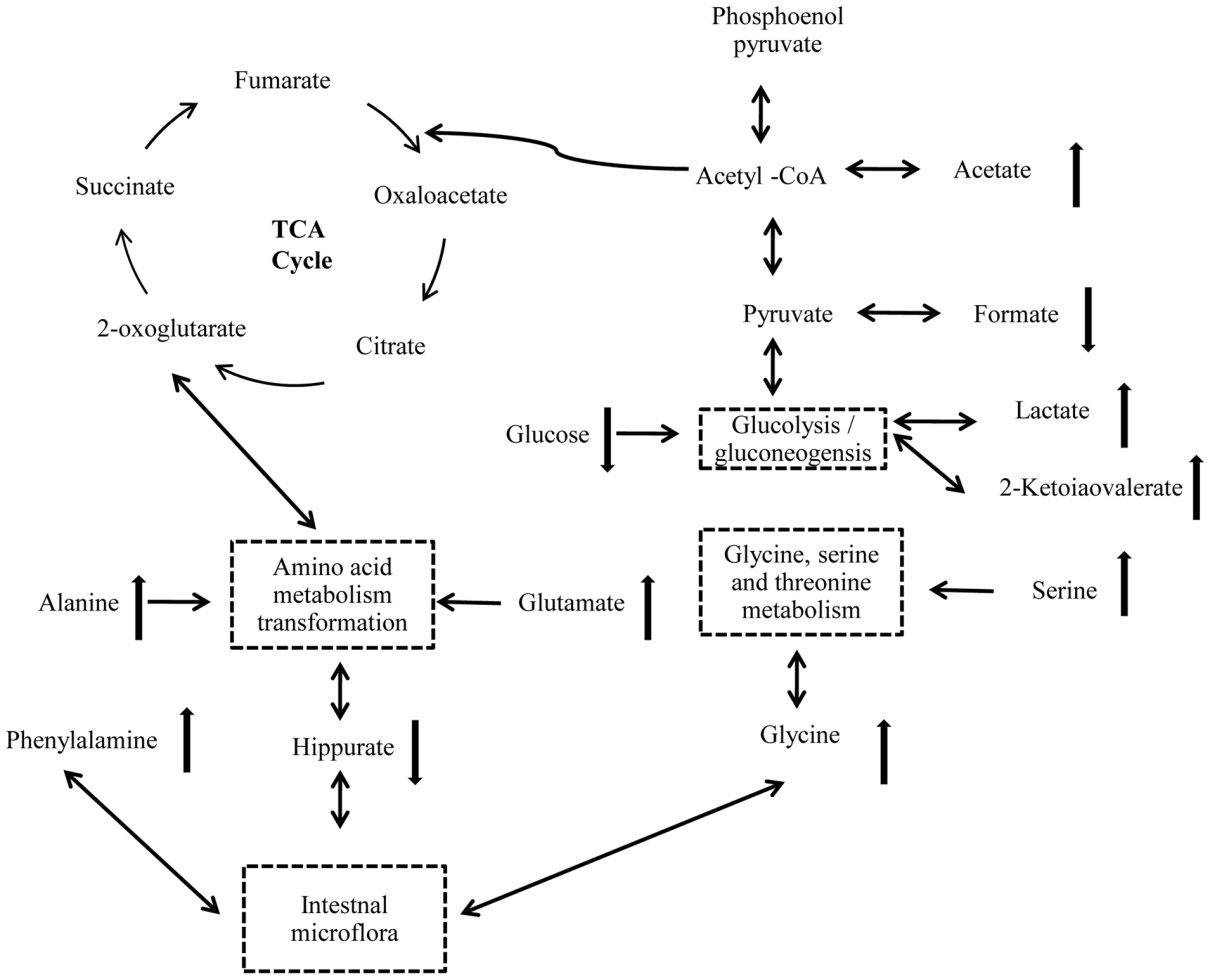
Altered metabolism pathways for the most relevant metabolic differences between rats treat with CR and control group. The network of the potential biomarkers changing according to the KEGG PATHWAY.

This is the first study researching the mechanisms of toxicity induced by CR using a holistic approach. The combination of NMR and GC-MS based metabonomics can comprehensively measure the metabolic responses of biological systems. In our study, urine and plasma samples were collected at multiple time points and a set of metabolites were assayed to identify differences in concentration. The results revealed that endogenous metabolites in urine showed time-dependent changes at different period of the experiment. After 14 days of CR administration, by ^1^H NMR, comparing with controls, we observed that 2-keto-isovalerate, lactate, alanine, N-acetylglycoprotein, glutamate, trimethethylamine and phenylacetylglulamine were markedly increased, while creatine, hippurate, trigonelline and formate were decreased. By GC-MS, alanine, L-proline, glycine, hisoindol, serine, glutamate and L-ornithine were distinctly elevated, while D-glucose and glycerol were decreased. We showed a simplified pathway map based on the metabolite markers identified by NMR and GC-MS (Figure 10). Figure 10 indicated that interference in intestinal microflora and disruption in energy metabolism dominated the altered biochemistry of CR treatment.

Hippurate is an acylglycine formed by the conjugation of benzoic acid with glycine in a reaction (acyl-CoA + glycine→CoA + N-acylglycine) catalyzed by glycine N-acyl trans-ferase [37], which is a metabolite of the amino acid metabolism refer to alanine, glutamate, glycine, serine and phenylalanine (http://www.kegg.jp). It has proved that intestinal microflora play an important role in the excretion of aromatic phenolic compounds. Hippurate, is generated from the conjugation of glycine and benzoic acid that is produced from aromatic amino acids by gut microbes and excreted into the urine [38]. In addition, it was reported that hippurate is a biomarker of breaking the balance of normal microorganisms, and that low levels of hippurate have been found in intestinal flora disturbance induced by GI damage [39]. Therefore, it is possible that CR treatment may correlate with a disturbance in the gut microbiota. The lower level of hippurate in urine at 14 days after CR administration may account for interference in amino acid metabolism. After 7 days of recovery, the level of hipprate was back to the value of pretreatment, which implied the gut microbiota may remold when CR treatment was stopped.

In addition, gut microbiota extensively catabolized protein and aromatic amino acids, including phenylalanine and tyrosine, to form phenylacetylglutamine and p-cresol sulfate [40]. As discussed the relationships between urine metabotypes and microbiota composition, phenylacetylglutamine displays positive correlation with Proteobacteria species, namely *Campylobacter, E. coli, Haemophilus, Pseudomonas, Serratia, Yersinia et rel* [22] . In this study, increased urinary level of phenylacetylglutamine may suggest that CR exposure enhanced the aromatic amino acid metabolism for disturbance in the gut microbiota. After 7 days of recovery, the concentration of Phenylacetylglutamine showed a similar turn back tendency, but it was not so obvious compared with hippurate. The urinary level of trigonelline, which produced via the conversion of S-adenosyl-methionine (SAM) to S-adenosylhomocysteine (SAH) by gut microflora [41], was also significantly decreased in CR treated rats, when compared to controls. This suggested that SAM was consumed and thus depleted in the trans-sulfuration pathway to produce glutathione that was depleted in CR treated group [41], [42]. Figure 9 showed the urinary level of trigonelline increased when stopped CR administration.

We observed a higher urinary level of lactate and 2-ketoisovalerate, and a lower level of serum glucose. These compounds were main metabolites involved in biochemical pathway of glycolysis or gluconeogenesis. Glycolysis is the process of converting glucose into pyruvate and generating small amounts of ATP and NADH. This is a central pathway that produces important precursor metabolites for the citrate cycle. Lactate is typically interpreted as a marker of anaerobic metabolism, and accumulation of it usually accounted for a higher energy demand in the biological system [43]. Under normal circumstances, lactate transform into pyruvic acid by Lactate Oxidase, then pyruvic acid participate in the citrate cycle (TCA cycle). The TCA cycle is an important aerobic pathway for the final steps of the oxidation of carbohydrates and fatty acids, simultaneously, produce ATP. ATP deficiency from mitochondrial dysfunction can be partially offset by increased glycolysis, and the AMP-activated protein kinase (AMPK) will promote glycolysis through phosphorylation and activation of phosphofructokinase-2 (PFK2), leading to increase levels of the glycolytic stimulator, fructose-2, 6-bisphosphate. In this study, increased lactate and 2-keto-isovalerate in combination with decreased glucose suggest that CR exposure may disturb the energy metabolism.

An acetic acid is usually fully ionized to acetate, a derivative of acetic acid [44]. In biochemistry, acetate and acetic acid are equivalent. When bound to coenzyme A, it becomes to acetyl-CoA, another important precursor metabolite, which is produced by oxidative decarboxylation of pyruvate and an important intermediate in the TCA cycle, central to the metabolism of carbohydrates and fats [43]. In this study, elevation of acetate also suggests that the metabolism of fats and carbohydrates was affected. The suppression of body growth in rats treated with CR may attribute to energy metabolism disorder. We supposed that CR-induced diarrhea leads to intestinal malabsorption of carbohydrates or fats, furthermore results in energy deficiency and energy metabolism disorder.

Besides interference in above biochemical pathways, there were significant differences in trimethylamine, ornithine and proline between CR and control groups. Trimethylamine (TMA), a product of choline degradation and an osmolyte that counteracts the effects of increased urine concentrations [45], [46]. The concentration of TMA in urine was elevated after 14 days of CR treated, suggesting suppression of the choline degradation pathway. Ornithine is one of products derived from biodegradation alkaloids [47], and increased concentrations of ornithine in serum may because of the primary components of CR are alkaloids [48]. Increased proline concentration in serum might be related to disturbance in arginine and proline metabolism (www.genome.jp/kegg).

### Conclusion

This paper integrated NMR and GC-MS metabonomics in combination with the multivariate statistical analysis (SIMCA-P) to discover the marker metabolites which contribute to the toxicity of CR treatment. The results showed the main toxic effect of CR is diarrhea, coupling with resistance of body growth. Through multivariate statistical analysis 12 marker metabolites from NMR and 8 ones from GC/MS have been found. Among those metabolites, hippurate, acetate, alanine, glycine and glutamate showed association to the gut dysbiosis, whereas others, such as lactate and 2-ketoisovalerate are likely to increase anaerobic glycolysis, meanwhile the CR-associated toxicity will recover when treatment was stopped. These results suggest that the main reason for the CR-associated diarrhea might be disturbance in the normal gut microbiota. This metabonomics approach may provide an effective way to study the alteration of gut microbiota, which is expected to find broader application in other drug-induced gastrointestinal reaction assessments.
